# Pulse Probiotic Superfood as Iron Status Improvement Agent in Active Women—A Review

**DOI:** 10.3390/molecules26082121

**Published:** 2021-04-07

**Authors:** Yolanda Victoria Rajagukguk, Marcellus Arnold, Anna Gramza-Michałowska

**Affiliations:** Department of Gastronomy Science and Functional Foods, Faculty of Food Science and Nutrition, Poznań University of Life Sciences, Wojska Polskiego 31, 60624 Poznań, Poland; yola.victoria.raja@gmail.com (Y.V.R.); marcellusarnold95@gmail.com (M.A.)

**Keywords:** pulse, probiotic, prebiotic, iron deficiency, women reproductive age, functional food

## Abstract

Active women or women of reproductive age (15–49 years old) have a high risk of suffering from anaemia. Anaemia is not solely caused by iron deficiency, however, the approaches to improve iron status in both cases are greatly related. Improving the iron status of active women can be done by dietary intervention with functional food. This review aims to provide insights about the functional food role to increase iron absorption in active women and the potency of pulse probiotic superfood development in dry matrices. Results showed that the beneficial effect of iron status is significantly improved by the synergic work between probiotic and prebiotic. Furthermore, chickpeas and lentils are good sources of prebiotic and the consumption of pulses are related with 21st century people’s intention to eat healthy food. There are wide possibilities to develop functional food products incorporated with probiotics to improve iron status in active woman.

## 1. Introduction

The dietary iron demand of active women or women of reproductive age (15–49 y) differs based on their reproductive status. According to WHO [1], approximately 29% of women in reproductive age suffer from anaemia. Although anaemia is not solely caused by iron deficiency, the approaches to improve iron status in both cases are greatly related. WHO [2] mentioned that improvement of iron status can be achieved by iron fortification and other micronutrients in food. Micronutrients such as vitamin A, zinc, folate and vitamin B12 are synergistically supportive to overcome anaemia by dietary intervention [3]. However, in most cases, dietary interventions are limited by the bioavailability of iron contained in the product.

Iron bioavailability can be increased by traditional household cooking, soaking, germination, and fermentation [4]. The synergic results between prebiotic and probiotic in the increment of haem iron in the human body have also been reported [5]. To meet the growing interest in functional food, the exploration of optional food matrices in carrying probiotic and prebiotic should be considered. Some roles of functional food including disease prevention, control of body function, reducing the risk of disease, reducing the effect of aging, and defending the body by reducing allergies and increasing the immune system [6]. This review aims to provide insights about the functional food role to increase iron absorption in active women and the potency of probiotic food development in dry matrices.

## 2. Active Women

**Figure 1:**
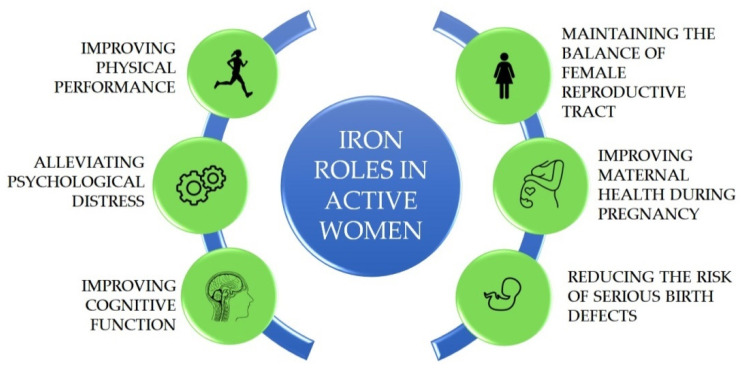
Iron roles in active women.

Active woman is defined as the woman being in her reproductive age between 15 and 49 years old. Throughout a woman’s lifetime, iron demands in the body would differ based on their reproductive status. In the reproductive age, it is important to maintain adequate iron stores in the body as the demand for absorbed iron would increase during pregnancy. Approximately 1000–1200 mg of iron are needed for a normal pregnancy, therefore an active woman should at least have ≥500 mg of body iron stores in the pre-pregnancy body [7]. During pregnancy, the body needs an increased amount of iron of around 700–850 mg as the depletion of iron stores takes place to support fetal growth [8]. After giving birth, the iron loss also occurs due to lactation via breast milk. The progressive iron depletion in the body without a balanced iron intake could cause anaemia with several health problems including fatigue, hair loss, dizziness, depression, decreased immune function, restlessness, and emotional instability [9]. The roles of iron in active women are depicted on Figure 1.

Up to 50% of anaemia cases are influenced by iron deficiency (ID) [9]. Anaemia is not only affecting the wellbeing and work performance of active women, but it also affects the living quality of the nation itself. Maternal anaemia has a higher risk of miscarriage, prematurity, and low birth weight of the baby. The generation of children that were born with maternal anaemia conditions would have impaired development and learning [10]. To a greater extent, reducing the prevalence of anaemia in active women would be the practical efforts to promote long-term nation’s health and productivity. Following the worldwide anaemia problem, the current target of WHO is 50% reduction in anaemia in women of reproductive age by 2025. One of the strategies to reach the target is the improvement of dietary diversity, for example, food fortification with iron and other micronutrients [2].

The latest data about the global prevalence of anaemia mentioned that approximately 38% of pregnant women, 29% of non-pregnant women and 29% of all women of reproductive age suffer from anaemia [1]. Additionally, more than 40% of women of reproductive age who suffer from anaemia are reported in the South East Asia region. There are several causes of anaemia, such as iron deficiency, inherited blood disorders, malaria, exposure to dangerous chemicals, schistosomiasis, and hookworm infections [11]. Among several factors causing anaemia, it is estimated that 50% of the reported prevalence of anaemia is caused by dietary iron deficiency [12]. The continuous situation of iron depletion could cause serious health problems which require comprehensive strategies to overcome the problem in the designed population. Iron deficiency can be overcome by dietary intervention, intramuscular injection, and supplementation, and an iron-rich diet involving the consumption of food that naturally contains a high level of iron, fortified food, or bio-fortified food [13]. Various fortification and dietary intervention studies have been reported to overcome iron deficiency and iron deficiency anaemia in women of reproductive age (Table 1). Another optional choice to fulfill the iron requirement is by iron supplement consumption, although the supplement has several side effects including abdominal discomfort, constipation, and nausea [14].

However, a study of the association between dietary iron intake and anaemia in India reported that providing iron fortification alone may not give a significant result of anaemia reduction. Besides iron fortification, combining iron-fortified food with other beneficial nutrients such as vitamin C and B12 should be considered [15]. Additionally, restoring the iron status of adolescents and adults with dietary intervention might give the same effect as consuming iron supplementation. A meta-analysis that compared the effects of dietary intervention and iron supplementation shows that no difference was observed in haemoglobin recovery between supplementation and dietary intervention in treating adolescents and adults [16].

## 3. Nutritional Behaviour of Women

Psychological and sociocultural factors may affect the nutritional behaviour of humans. Women generally represent a higher awareness of healthy food consumption than men. A study among Caucasian adults (12–75 years old) about the gender differences in taste and food habits shows that women eat a higher amount of whole grain food and lower processed meat than men [24]. Furthermore, the study mentioned that women missed more meals than men, although women are more frequently eat uncontrollably even if they are not hungry. Healthy snacking correspond to the maintenance of healthy body weight. In response to the women’s dietary pattern, healthy snacking has the potential to take part in the women’s habitual diet. Nevertheless, a study reported that there is a great gap between the shopping intention and shopping behaviour of women when they face a situation to choose food based on their healthiness or tastiness. The result showed that those who intend to shop healthily but commonly fail to do so are influenced by their behaviour to categorise food based on the tastiness factor [25]. It is important to design tasty food along with its high nutritional value.

As a woman gets older, there are some significant changes within the consumption behaviour and cravings. A study reported that women aged more than 25 years old have a strong relationship with sweet food cravings and emotional eating [26]. Meanwhile, the data showed that younger women are related to the consumption of fatty and sweet food to fulfil their cravings. Older women tend to suppress their food cravings as its consumption is associated with the increase in BMI. As one of the results of the unbalanced diet, excessive BMI may promote obesity. Additionally, obesity can influence the lack of duodenal iron absorption which eventually leads to iron deficiency and anaemia [27]. Some programs are introduced to overcome anaemia by nutritional intervention, such as vitamin A supplementation, food or condiment fortification (with vitamin A, iron, zinc, folate, vitamin B12, etc.), and dietary diversity [3]. The odds of anaemia can be reduced by 8% after the increased intake of dietary iron by 10 mg/d. When vitamin B-12 and C are consumed, the odds of anaemia can be reduced by 1.5% [15].

## 4. Iron Deficiency in Women

Iron deficiency is the term used to describe the condition when the iron stores in the body are depleted without the presence of anaemia. In iron deficiency, only the amount of iron-specific tissue/organ is depleted, while the amount of total body iron is still maintained because of the changes of body iron distribution [28]. Progressive iron deficiency may result in iron deficiency anaemia. Some symptoms of iron deficiency are lack of muscle strength, physical performance, and cognitive impairment (Table 2). According to Lanzkowsky [29], iron deficiency is caused by several aspects such as deficient intake, inadequate absorption, increased demand of the body (growth, adolescence, pregnancy), blood loss, impaired absorption (malabsorption disease, celiac disease, chronic gastritis), inadequate presentation to erythroid precursors, and abnormal intracellular transport or utilisation. The author further added that there are three stages before iron-deficiency anaemia occurs; (a) iron depletion as the iron stores in tissue decrease; (b) iron-deficient erythropoiesis, the serum iron amount becomes less without the change of hematocrit; (c) iron-deficiency anaemia, detected by the abundant amount of erythrocytes after the lack of availability of iron.

### 4.1. Dietary Intake of Iron

Iron is one of the important micronutrients required in energy production, oxygen transport and the functional component of haemoglobin and myoglobin in the body [36]. In order to achieve balanced metabolism and oxygen transfer, the level of iron must be sufficient in the body. Dietary iron consists of haem and non-haem iron. Haem iron refers to iron from the animal, while non-haem is for the rest of iron excluding animal-originated iron [37]. Dietary reference values (DRV) for iron are determined based on several variables related to the amount of iron loss from the body and the iron absorption capability from the body of the representative group [38]. The DRV of female adolescents aged 12–17 years old, premenopausal, and postmenopausal women aged ≥18 years old are mentioned as follows 13 mg/day; 16 mg/day; and 11 mg/day. Furthermore, EFSA also stated that the DRV of pregnant and lactating women follow the same DRV of premenopausal women, which is 16 mg of iron/day. According to Guo et al. [39], the demand for iron in pregnancy would increase from 0.8 mg/day to 7.5 mg/day in the third trimester to support fetal growth. However, the increasing demand for iron will also be followed by the increase in iron absorption efficiency. Therefore, with the same DRV with premenopausal women, it was assumed that the enhanced iron absorption and the mixed diet in the later stage of pregnancy could compensate for the iron losses in the body [38].

In contrast to the DRV of iron from EFSA, a study about the iron intake in women of reproductive age from 29 countries in Europe reported that up to 97% of women have a dietary iron intake below 15 mg/day [7]. This low iron intake might be influenced by the low intake of haem iron and unbalanced amount of inhibitor and enhancer of iron in the diet.

### 4.2. Iron Losses

Iron requirements are determined to compensate for the estimated iron losses in the body. The higher the estimated iron losses, the higher DRV requirement. According to Ghosh et al. [40], total iron loss in women was calculated by the sum of menstrual and basal iron loss. The estimation of basal iron loss was obtained by measuring the iron loss in hair, nail, gastrointestinal tract, urinary and other exogenous sources than hair, and nails. The sum of the estimated basal iron loss is 0.93 mg/day. Meanwhile, estimating the iron loss from the menstrual cycle depends on some factors such as age, reproductive status (middle-late reproductive age, and early-late menopausal transition), and food habits of the population [41]. In physically active individuals, iron loss occurs along with the increase in haemolysis during endurance training. Iron losses in a physically active population occurred through gastrointestinal bleeding, sweat, and hematuria [42].

### 4.3. Indicators of Iron Status

Some indicators commonly used to determine iron status in the body are haemoglobin concentration, serum ferritin, soluble transferrin receptor (sTfR) and total body iron [43]. According to Pfeiffer and Looker [44], serum ferritin is a sensitive indicator before the body iron stores are depleted. After they are depleted, serum sTfR is preferable to use as an indicator of body iron stores. Additionally, haemoglobin concentration is a reliable indicator that represents iron deficit, especially in the body that suffers from iron deficiency anaemia. Anaemia condition was defined when the body status met the indicators’ requirement in Table 3. Meanwhile, the safe cut value of serum ferritin for iron deficiency is 30 µg/L [41].

### 4.4. Iron Absorption

In the body, iron exists in erythrocytes as the haem compound of haemoglobin and in the muscle cells as myoglobin [45]. Iron absorption mainly occurred in duodenum and proximal jejunum. Iron must present in the ferrous state (Fe^2+^) or bounded with protein such as haem, in order to be absorbed. The absorption of haem iron is four times more efficient than non-haem iron [46]. Meanwhile, non-haem iron exists as the insoluble ferric form (Fe^3+^) after it is released from other food components. The body will absorb non-haem iron only if it is reduced to ferrous iron (Fe^2+^). The transformation of Fe^3+^ to Fe^2+^ occurs by the presence of duodenal ferrireductases. The presence of other reducing agents in diets such as ascorbic acid, other organic acids, and amino acids (cysteine and histidine) could stimulate endogenous gastric acid production which influences iron absorption [47].

Some factors influencing the iron absorption rate include the valence of iron, chemical form of iron, the presence of competitors, enhancers, inhibitors from food, and the individual iron status [41]. According to Alaunyte et al. [37], the iron absorption will be reduced when the body has an adequate level of iron. Meanwhile, during iron deficiency situations, even non-haem dietary iron becomes an essential source of absorbable iron. Some competitors that influence iron absorption are lead, cobalt, strontium, manganese and zinc [48].

### 4.5. Iron Bioavailability

The term bioavailability means the ability of substances to be absorbed in the human body. The absorption of iron depends on the type of iron (haem or non-haem iron), the presence of enhancers such as vitamin C, certain organic acids, and inhibitors such as phytates, phenolic compounds, and divalent ions (zinc, manganese, and calcium) present in the diet (Table 4) [37]. Other than those factors, the amount of iron ingested, the meal composition, and the time between meals are also important factors related to bioavailability [49]. Coad and Pedley [47], conclude that to promote the better absorption of iron, consuming iron with ascorbic acid-rich sources and avoiding polyphenol as well as other inhibitors consumption are needed. The addition of prebiotic mix containing inulin and fructooligosaccharides (FOS) is also reported to boost the bioavailability of haem iron in the human body [5].

Among all enhancers, ascorbic acid (AA) is the most effective enhancer for non-haem iron absorption [49]. This enhancing effect has no impact on haem iron. A study on the effectiveness of AA as an enhancer in the presence of an inhibitor has been reported [50]. The authors mentioned that in low to medium levels of inhibitor AA, 2:1 of molar ratio between AA and iron need to be added. Meanwhile, in a high-level inhibitor, it is necessary to add 4:1 of molar ratio. AA can reverse the inhibition of phytic acid (PA), sodium oxalate (SO), and sodium silicate (SS) [51]. The increment of ferric sulphate iron absorption was observed in the ratio of 5:5:1 AA:PA:Fe, 3:5:1 AA:SO:Fe, and 5:5:1 AA:SS:Fe. Other reported weaker enhancers than ascorbic acid are lactic, citric, malic, and tartaric acid [50]. Besides acid groups, the study of the mixture of ferric sodium EDTA and ferric sulfate shows the effectivity of the salt to enhance iron absorption in the presence of phytate [52].

## 5. Pulse as a Diet Modulation Tool

Pulse is defined as the edible dry seed of legume. There are 11 classifications of pulse including common dry beans, dry broad beans, dry peas, chickpeas, dry cowpeas, pigeon peas, lentils, bambara beans, vetches, lupin, pulse nes (other types of pulse that do not belong to any categories), pulse flour and pulse bran [58]. According to Rawal and Navarro [59], some major categories of pulses that are consumed in large quantities in the world are chickpeas, lentils, common bean, dry peas, dry cow peas and pigeon peas. Pulses are considered as a part of a healthy diet as they are naturally low in the glycemic index (GI) which would be a suitable diet for people with type II diabetes [60]. Pulses are also a good menu option for a weight loss diet, because the dietary fibre and complex carbohydrates from pulses would slowly be digested and it helps to make people feel full longer. A recent study reported that a diet with high pulses intake causes a greater body weight loss compared to a diet without pulses [61].

Furthermore, it contains a rich amount of essential nutrients and vitamins which come along with various health benefits. Several anti-nutritional compounds of pulses such as trypsin inhibitor, glycosides, phytates, and tannin can be eliminated through the cooking process. The elimination of these anti-nutritional compounds is considered important because their presence causes bloating, abdominal discomfort, and affects mineral absorption [62]. Abdominal discomfort is caused by the presence of non-digestible components in pulses such as oligosaccharides, resistant starch, and non-starch polysaccharides. These antinutritive components can be reduced by hot extrusion at 160 °C for chickpea flour [63], traditional and microwave cooking, soaking, germinating, and fermentation [64].

Pulses are also an important source of some essential minerals such as iron, zinc, selenium, phosphorous, and potassium [65]. In developing countries, the pulse is mainly consumed due to its nutritional value and the low cost of the product [66]. Despite the presence of minerals in pulses, the bioavailability of these compounds is low. Whole pulse grains contain a higher amount of mineral than decorticated grains. Compared to other crops, pulses contain higher amounts of calcium than wheat and higher potassium than cereals. The amount of minerals present in the pulse depends on the type of soil and fertiliser that is used to grow the pulse [67].

### 5.1. Production and Consumption Trend of Chickpeas and Green Lentils

**Figure 2:**
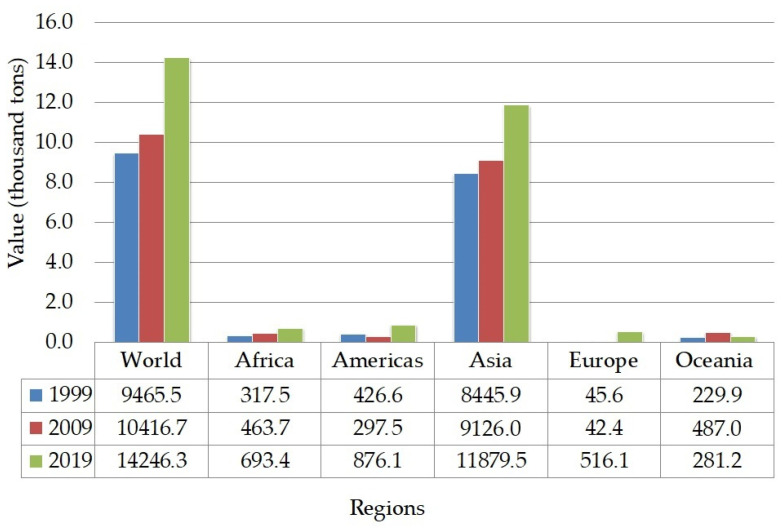
The trend of chickpeas production [68].

FAO (Food and Agriculture Organization of the United Nations) predicted that the global consumption of pulses will be increased to 96.5 million tonnes in 2025 [59]. The projection of global consumption increment is also supported by the production growth of chickpeas from 1999 to 2019 (Figure 2). The highest quantity of chickpeas is produced in the Asian region, mainly South Asia.

Additionally, U.S. dietary guidelines recommend consuming 1–1.5 cups (192 g) of legumes a week. The dietary recommendation is followed by the increasing trend of chickpeas and hummus consumption in U.S from 2005 to 2016. A study about the nutritional status of people who consume chickpeas and hummus in U.S. population showed that they are less likely to suffer from metabolic syndrome [69]. The authors further stated that hummus and chickpeas are mostly consumed by the female consumers during lunch, dinner, and snacking occasions. According to Merga and Haji [70], the world’s chickpeas production is predominated by 80% desi type instead of kabuli which is more preferable for the market outside South Asia. Seed coats of desi type are more pigmented and thicker than kabuli. In South Asia, desi type is often processed into “dal”, it is often used as the main ingredient in soup. Dal is the split cotyledons of desi type after the seed coat decortication.

**Figure 3:**
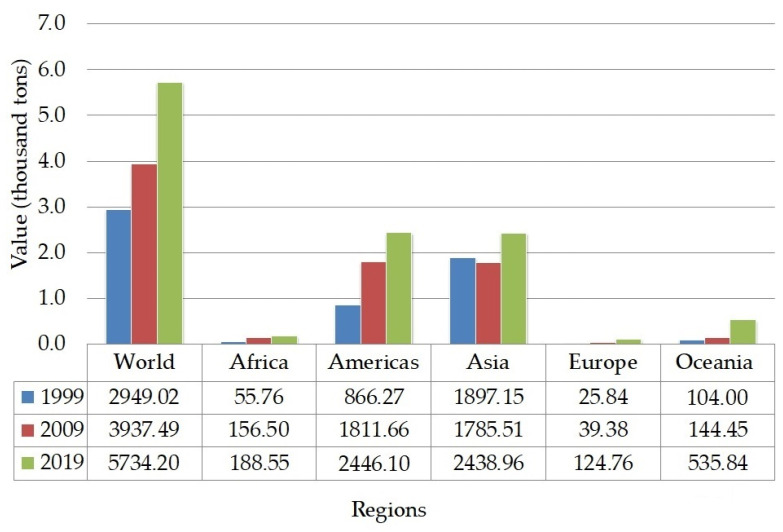
The trend of lentils production [68].

The production of lentils (Figure 3) showed a significant increment within the past 20 years. The highest amount of lentil production was recorded in American and Asian regions, specifically in Canada and India [68]. The production mainly covered two types of lentils, such as red and green. The higher demand for red lentils in South Asia makes the world’s production slightly shifted to produce a greater amount of red lentils than green lentils. Green lentils are mainly consumed in West Asia, North Africa, and Europe [59]. The trend of consuming healthy snacks has driven the food industry to incorporate lentils into various food products, such as substituting cereal-based flour with lentil flour in baked and snack products [71]. Meanwhile, in traditional Asian dishes, lentils are consumed as the main ingredient in curries, dal, and soup.

### 5.2. Characteristic of Pulses and Their Functional Properties

Chickpeas (*Cicer arietinum* L.) is the seed from the *Fabaceae* family that originated from the south-eastern part of Turkey [72]. Nowadays, chickpeas are widely consumed and produced in India with 67% share of global production followed by Australia, Pakistan, Myanmar, Turkey, Ethiopia, and Iran [59]. Chickpeas are classified into two types of seeds, which are desi and kabuli. Desi has a thicker pericarp with darker pigmentation, mainly cultivated in a warm environment such as Asia and Africa. Kabuli has a slightly bigger seed than desi with beige color, grown in the Mediterranean region, North Africa, Europe, South and North America [73].

Protein and carbohydrate are the major components in the chemical composition of chickpeas (Table 5). The digestibility of protein from chickpeas varies within the range 49–89.1% [63]. The authors explained that the digestibility of protein from chickpeas can be increased by fermentation, germination, and cooking. Processing the chickpea seeds by cooking results in the increment of fibre, total carbohydrate, and total resistant starch content [74]. Resistant starch is categorised as the insoluble part of carbohydrate that naturally occurs in legumes, cereals, whole grains, beans, etc. [75]. In the digestive tract, resistant starch cannot be hydrolyzed due to the lack of α-galactosidase in the human body. The resistant starch is accumulated in the large intestine and is fermented by the colonic bacteria. The presence of resistant starch from chickpea influenced the growth of Bifidobacteria in the colon. In the other words, resistant starch that exists in chickpeas has a significant role as prebiotic for colonic bacteria [76]. Meanwhile, the soluble fibre in chickpeas will be digested slowly in the colon.

The fat content of chickpeas is higher than lentils (Table 5) and other pulses such as red kidney bean, mung bean, pigeon pea, etc. [77]. The authors further explained that chickpeas also contain a higher amount of linoleic acid (51.2%) and oleic acid (32.6%) compared to other pulses. Despite the high nutritional content available in chickpeas, it contains several anti-nutritional components such as tannin (0.4–0.8%), trypsin inhibitor (3–9 trypsin inhibitor units per mg), phytic acid (0.28–1.6 g/100 g), saponin (0.9 mg/100 g) and haemagglutinin (6.2 HU/mg) [4,76]. The presence of tannin and phytate can strongly affect mineral’s bioavailability, such as iron, calcium, and zinc (Table 6). Mineral’s bioavailability from chickpeas can be improved by pre-treatment such as soaking and germination. Soaked (24 h) and germinated (24 h) chickpeas are proved to have a better mineral bioavailability than raw chickpeas [78]. Germination improved the bioavailability of iron by breaking down phytate, tannin, and other anti-nutritional compounds through enzymatic activity.

Due to their nutritional and bioactive properties, chickpeas are utilised in various food and beauty products for various purposes. Chickpeas in powder form are used as facial masks, added components in anti-dandruff products, substitute of coffee, and addition to food products [63]. Besides providing basic nutrition, consuming chickpeas may provide additional health benefits such as reducing the risk of obesity, diabetes type II, cardiovascular disease, colorectal cancer, and improving bowel health [79]. Consumption of chickpeas was proved to increase the frequency of defecation with softer stool consistency compared to a regular diet with lower pulses intake [64].

### 5.3. Lentils

During the recent decades, lentils (*Lens culinaris*) have gained a reputation as a healthy and functional food option. It is proved by the increasing amount of the world’s lentil production during the last three decades. In 2014, FAOSTAT reported that Canada is the largest producer of lentils, followed by India, Turkey, Nepal, and the United States of America. Lentils can be distinguished into five major types of colour which are green, red, small brown, French green, and black lentils [71]. The darker colour in pulses indicates the higher phenolic and antioxidant content than the fair coloured pulses. According to DellaValle et al. [82] the iron bioavailability in green lentils is significantly higher than in red and green lentils although phytic acid concentration of green and red lentils are in the same range. Furthermore, the author mentioned that the phytic acid concentration of pulses depends on the presence of phosphorus in the soil. Although it is considered an anti-nutritional factor, soil phosphorus is needed for plant metabolism as well as for human health.

Lentils contain an abundant amount of protein compared to chickpeas (Table 5). The predominant protein in lentils is globulin and albumin [83]. The utilisation of protein and carbohydrate from lentils is limited to the presence of anti-nutritional components including trypsin inhibitor which is able to inactivate the digestive enzyme, and also phytic acid and tannin that decrease mineral absorption [84]. As one of the sources of minerals for people with a vegetarian diet, lentils have a great amount of iron although their bioavailability is low. A study reported that the absorbed amount of iron from lentils is significantly lower than the absorption of iron in the form of ferrous sulphate for women with poor iron status [85]. Some of the efforts to increase iron absorption from lentils consumption are iron fortification of lentils with NaFeEDTA [86] and processing the raw lentils with dehulling, germination, and cooking [84]. The iron absorption increase as the amount of phytic acid, tannin and other antioxidant metabolites (gallic acid, catechin, and quercetin) decreases after the cooking process. The amount of phytic acid present in lentils is 0.21–1.51 g/100 g [4].

Various forms of lentils can be applied for different kinds of purposes in food. The whole grain of lentils is usually used as salad, soup, snacks, and canned food mix. Meanwhile, lentil flour can be utilised as a partial substitution of wheat flour in baked products. According to Chelladurai and Erkinbaev [71], the addition of lentil flour aimed to provide better nutritional content such as protein and dietary fibre. However, the greater incorporation of lentil flour to the baked product does not positively impact consumer acceptability. Portman et al. [87], reported that the optimum addition of lentil flour to the baking mix containing wheat flour and additional gluten is from 5% to 20% as it affects the physical properties of the products. Besides bakery, lentils flour is also incorporated in pasta and noodles as one of the nutritious gluten-free products [88]. Compared to other pulses, lentils contain a higher amount of polyphenols that provide various health-promoting effects such as protection against diabetes, obesity, cardiovascular diseases (CVD), and cancer [83,89].

### 5.4. Increasing Bioavailability of Iron in Pulse

According to Sumengen et al. [90], phytate works as a highly negative charged ion, this explains the low bioavailability of minerals (Ca^2+^, Fe^2+^/^3+^, Zn^2+^, and Mn^2+^) in the presence of phytate. Some traditional ways to increase the bioavailability of mineral from pulses in household cooking are dehulling to reduce polyphenol, germination, fermentation, and soaking for phytate reduction [4]. According to Whiting et al. [91], the soaking process activates the phytase activity, thus the reduction in phytate took place. Factors influencing the reduction in phytic acid are the time of soaking from 4 h to 25 h, cooking time and the density of the cotyledon in pulses [92]. Furthermore, the amount of water penetrating the seeds during cooking depends on the density of cotyledon. The presence of boiling water inside the seeds helps the formation of an insoluble complex between phytic acid and ion, resulting in the reduction in phytic acid [67]. Hence, adequate time in soaking and cooking is important to control with an increment of iron bioavailability. Shi et al. [92] reported that soaking several types of pulses such as chickpeas, lentils, fava beans, peas, beans, and soybean for 4 h does not give a significant impact on phytic acid reduction although longer soaking time does not necessarily provide a better result.

Another method to increase the bioavailability of iron in pulses is by canning. Some steps included in canning are soaking, blanching, the process of canning, cooking, and autoclaving. A study about the comparison between household cooking and canning of the bioactive composition of pulses has been done. The result showed that the canning reduces 24% of the phytate contents in chickpeas, which is lower than household cooking [93]. However, excessive exposure to heat during canning could also cause greater losses of some compounds such as water-soluble micronutrients, protein, and fibre. New technologies such as cold plasma, pulsed electric field, UV, irradiation, and ultra-sonification can also be utilised as the pre-treatment strategies to decrease anti-nutritional components [92].

In developing countries, the effort to increase iron bioavailability has been done by applying biofortification to several of the most consumed pulse crops. According to Jha and Warkentin [94], the process of improving the nutritional status of plant-based food by applying genetic engineering techniques in plant breeding is defined as biofortification. After 18 weeks of consuming iron biofortified beans, the total body iron and haemoglobin in Rwandan women have been improved [18]. The iron-biofortified beans are naturally high in iron and are designed to have lower phytic acid [95].

Increasing the bioavailability of iron by reducing phytic acid can also be done by using other phytase sources such as from animals, plants, and microorganisms. Phytase produced by microorganisms is much more stable at higher temperatures and pH which is preferable for industrial usage [4]. Furthermore, phytase from microorganisms is preferable because it provides an optimal environment for enzymatic degradation during natural fermentation. Among phytase-producing microorganisms, lactic acid bacteria (LAB) are labeled as GRAS (generally recognised as safe) in food industry applications. In vitro studies of the effectivity of phytase-producing LAB to increase the solubility of iron and hydrolysing phytate has been done in various food media such as sourdough, soy milk, fruit juice, cereal-based beverage and fermented vegetables [96]. Some of the commonly used phytase-producing LAB are *Lactobacillus* sp. and *Bifidobacterium* sp. [97]. Vonderheid et al. [98], mentioned that *Lactobacillus plantarum* 299v consumed through supplementation, performs a significant role to increase non-haem iron absorption and iron status in a healthy European woman.

## 6. Probiotics as Allies in Nutrition

Probiotics are defined as living microorganisms present in an adequate amount that significantly contribute to the health benefit of the host. Consuming probiotics is not limited to the fermented product or another food medium in liquid form. Some studies applied probiotics in the form of encapsulated powder to the snack bar or energy bar [99,100,101]. Besides encapsulated probiotic powder, a study shows a possibility to use the resulting probiotic culture directly after biomass separation. Kavitha et al. [102] reported that the range of viable count from probiotic cultures (*Lactobacillus acidophillus, Lactobacillus delbrukii and Streptococcus acidophillus*) are from 1 to 3.5 × 10^8^ CFU/m.

The ability of probiotics to produce phytase is important to consider during the selection of probiotic. Some notable LAB for highly producing phytase ability are *Weisella kimchii*, *Lactobacillus brevis*, *Bacillus subtilis* and *Lactobacillus plantarum* [103,104]. Some factors influence the phytase activity in probiotic, such as bacterial viability, optimal pH, accessibility of phytate, presence of inorganic phosphate, and other organic acids. A higher amount of minerals such as calcium and phosphate can bind the enzymatic active site, resulting in the inhibition of phytate degradation [104].

The most common LAB found in fermented food is *Lactobacillus plantarum*. *Lactobacillus plantarum* isolated from Turkish fermented food (Shalgam) was reported to have the capability of producing a significant level of phytase. The molecular weight of extracellular phytase was estimated to be 46 kDa, while intracellular phytase is 36 kDa [90]. The optimum pH and temperature of phytate-degrading activities of several Lactobacillus plantarum strains are in the range from pH 5.0 to 7.5 and from 50 to 70 °C [105]. Furthermore, a study reported that the fermentation of *Lactobacillus plantarum* 299v proved to increase the mineral accessibility in pseudocereals and grains flour [106]. A study about the effect of multispecies probiotic supplementation on the improvement of iron status on rats reported that the minimum daily dose of probiotic supplement is 1 × 10^10^ CFU [107].

According to Skrypnik [107], multispecies probiotic supplementation causes a significant increment of duodenal iron levels. The authors reported in the first stage ellagic acid being converted to urolithin A by probiotic bacteria. As ellagic acid is no longer able to bind Fe^3+^ after the conversion took place, Fe^3+^ is ready to react with other substances. After the conversion, p-hydroxyphenyllactic acid excreted by *Lactobacillus* sp. will increase the amount of absorbable iron by reducing Fe^3+^ to Fe^2+^. Besides increasing iron absorption, probiotics also need to have high bacterial survival ability during food storage and transit through the gastrointestinal system. In a food matrix, the existence of prebiotics is important to support probiotics’ viability.

Prebiotic dietary fibres are the carbon sources for the primary and secondary fermentation pathways in the colon by selectively fermenting bacteria, which provides the health promoting effects of the host [108].

Prebiotics such as oligo and polysaccharides including inulin, fructooligosaccharide (FOS), and galactooligosaccharide (GOS) were reported to have protectants’ ability towards lactic acid bacteria. The prebiotic role as protectants has been shown during the probiotic starter growth, the survival in food matrices, and the gastrointestinal tract [109]. However, Sidhu et al. [110] reported the addition of chickpea flour as a prebiotic does not give any significant impact on probiotic viability during storage. The authors further mentioned that chickpea flour’s role as a protectant appears during the presence of gastric and intestinal juices. Besides the role as a protectant, the synergic effect between prebiotic and probiotic in improving iron absorption is also studied through some possible mechanisms. According to Arnold et al. [111], the fructans are fermented by the probiotic or gut microbes, then the fermentation products, such as organic acids, decrease the pH in the colon, and could increase the mineral absorption. The decreasing pH condition helps the conversion of iron from ferric form (Fe^3+^) to ferrous form (Fe^2+^), which is more readily bioavailable [112]. Furthermore, when the prebiotics are ingested, osmotically active sugars produced via fermentation can increase the passive absorption of metals, such as iron [112,113]. Short chain fatty acids (SCFAs) such as acetate, propionate, and butyrate are also generated by gut microbiota through the fermentation of prebiotics. The SCFAs can enhance the surface area for iron absorption through the proliferation of colonic epithelial cells [114]. This is supported by a study which reported the prebiotics effect (inulin, FOS, GOS, and lactulose) on the iron absorption and iron status of anaemic rats [115]. It was reported that rat groups with FOS or GOS treatments had significantly higher iron absorption, and the SCFAs concentration was increased among the treatment groups with prebiotics fermentation, carried out by probiotics. This phenomenon concluded that the prebiotics addition increases the absorption of iron in anaemic rats.

The incorporation of probiotic in food is not limited to fermented dairy, fruit, and vegetable products. Various types of food outside fermented products have been developed as probiotic carriers (Table 7). These food matrices offer attractive alternatives for food consumers who want to reduce their dairy consumption but still want to get the benefit of probiotic food. Additionally, dry food incorporated with probiotic possesses a long shelf life at ambient temperature and results in the reduction in logistic cost during storage [116]. Dry food incorporated with probiotic has shown a promising capability as a probiotic carrier in the functional food market. Some potential challenges related to probiotic food development in dry food matrices are advanced drying techniques for the probiotic cell, maintaining the probiotic’s viability, as well as choosing the right food matrices to promote significant synergism with probiotics and prebiotics.

## 7. Conclusions

**Figure 4:**
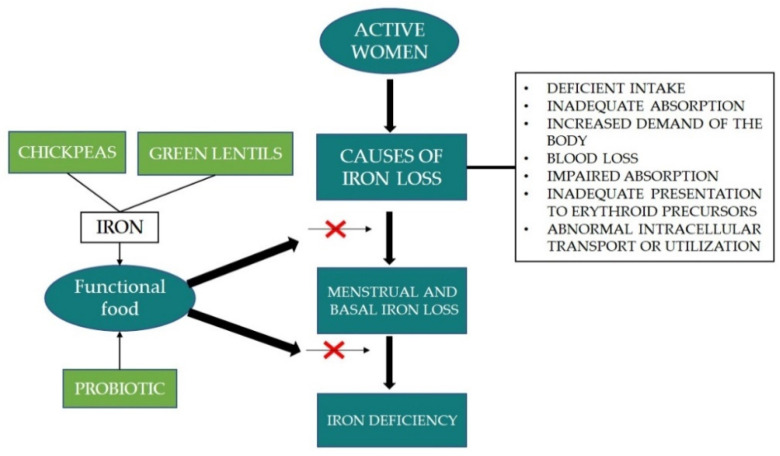
Functional food as iron status improvement agent in active women.

Scientific observations indicate that total iron loss in women is caused by inadequate or impaired absorption, increased demand of the body, blood loss, abnormal intracellular transport or utilisation and inadequate presentation to erythroid precursors. In addition to these factors, the amount of iron loss is also influenced by age, reproductive status, as well as food habits. The process of iron bioavailability depends on many factors, e.g., the type of iron, the presence of vitamin C or certain organic acids, but also the presence of inhibitors such as phytates, phenolic compounds, and divalent ions present in the diet. Improving the iron status of active women can be done by dietary intervention with a functional food (Figure 4). Results showed that the beneficial effect of iron status is significantly improved by the synergic work between probiotic and prebiotic. Therefore, chickpeas and lentils are good sources of prebiotic and the consumption of pulses is related to 21st century people’s intention to eat healthy food. This trend has prompted the food industry to include pulses, especially lentils in the recipes of foods and snack foods, where cereal flours are replaced with lentil flour.

The great advantage of pulses is the possibility of moderating their properties through the use of selected culinary techniques. Targeted processing of lentils results in the increment of total carbohydrate, fibre, and resistant starch content. Resistant starch, the insoluble part of carbohydrate naturally occurring in legumes, cannot be hydrolyzed in the digestive tract of human body due to the lack of α-galactosidase. Therefore, resistant starch found in chickpeas plays a significant role as a prebiotic for colon bacteria, and at the same time the soluble fibre fraction is slowly digested in the colon. As a result, prebiotics are fermented by probiotics or intestinal microbes, and the products of this process, such as organic acids, lower the pH in the colon and thus increase the absorption of minerals. Modern technologies provide wide opportunities for the development of functional food products incorporated with probiotics to improve iron status in active women. However, further studies should be conducted to see the interaction between probiotic and phytic acid from pulses in iron absorption, especially in women’s bodies.

## Figures and Tables

**Table 1:** Studies about dietary intervention and its effect on iron status improvement.

No	Region, Country	Population	Diet Duration	Dietary Intervention	Notes	References
1	Gothenburg, Sweden	Women (mean aged 24 )	84 days	Blood-based crisp bread	The increase in body iron is not significantly different with the group receiving non-haem iron supplementation. Dietary-based treatment with haem iron is efficient to improve iron status.	[17]
2	Huye, Rwanda	Women (aged 18–27)	128 days	Iron biofortified beans	Increase in haemoglobin (3.8 g/L), log serum ferritin (0.1 log µg/L) and body iron (0.5 mg/kg)	[18]
3	West Bengal, India	Women (aged 18–55)	10 month	Double fortified salt with iodine and iron	Salt double-fortified with microencapsulated ferrous fumarate and potassium iodate resolves iron deficiency and low body iron in the population.	[19]
4	Chester, UK	Women (aged 19–49)	8 weeks	Iron fortified breakfast cereal and vitamin D3 supplement	The improvement of haemoglobin concentration and haematocrit levels were observed in women with low iron stores.	[20]
5	Madrid, Spain	Women (aged 18–35)	16 weeks	Iron and vitamin D fortified flavoured skim milk	Iron and vitamin D-fortified flavoured milk slightly enhances erythropoiesis and iron status compared to milk fortified solely with iron.	[21]
6	Auckland, New Zealand	Women (aged 18–44)	16 weeks	Iron fortified breakfast cereal with kiwi or banana	Consumption of iron-fortified breakfast along with ascorbic acid-, lutein-, and zeaxanthin-rich fruit can improve the iron status in women.	[22]
7	Madrid, Spain	Women (aged 18–35)	16 weeks	Iron pyrophosphate and vitamin C-fortified fruit juice	Iron pyrophosphate and vitamin C-fortified fruit juice can improve the iron status in women.	[23]

**Table 2:** Health conditions associated with iron deficiency in active women.

Health Issues	Notes	References
Fatigue, anger, tension, depression, and emotional instability	Young women aged 18–22 years old with iron deficiency showed higher proportion of neurotic tendencies	[9,30]
Hair loss	Premenopausal female pattern hair loss patients (FPHL) showed lower serum ferritin concentration than control group	[9,31]
Restlessness	Non-anaemic iron deficiency women showed a higher severity of restless legs syndrome (RLS) and tiredness during the day than patients without iron deficiency.	[9,32]
Lack of muscle strength and physical performance	Women aged 18–45 years old with non-anaemic iron deficiency had significantly lower VO_2_ at the ventilatory threshold than the control group. The study further mentioned that women with iron deficiency spend more time in sedentary behaviour than light physical activity	[29,33]
Cognitive impairment	Women aged 20–32 years old with non-anaemic iron deficiency reported to have lower endurance during a cognitive-demanding task.	[29,34]
Adverse pregnancy outcomes (prematurity, miscarriage, low birth weight of the baby)	Maternal iron deficiency could affect the development of fetal brain	[10,35]

**Table 3:** Indicators for iron status interpretation: cut values for anaemia.

Indicators	Amount
Haemoglobin	<120 g/L
Serum ferritin	<15.0 µg/L
sTfR	>8.3 mg/L
Total body iron (Cook’s equation)	<0.0 mg/kg

Source: [43].

**Table 4:** Iron deficiency and anaemia in relation with other vitamin and mineral deficiency.

Micronutrients/Food Components	Notes	References
Vitamin A	Vitamin A has shown the role in iron homeostasis through hepcidin. A cross-sectional study reported that retinol-binding protein that represents vitamin A has a significant correlation with haemoglobin concentration in the human body.	[53,54]
Vitamin D	Hepcidin is reported to be elevated along with vitamin D deficiency. A high level of hepcidin causes the amount of serum iron to drop, which might lead to iron deficiency and anaemia.	[55]
Vitamin B_12_ and B_9_	Vitamin B_12_ and B_9_ deficiency are recognised by the elevated levels of homocysteine. There is a significant association between homocysteine level and biomarkers of iron deficiency and anaemia (haemoglobin, serum ferritin, and hematocrit).	[56,57]
Zinc	Zinc acts as a catalyst in haem metabolism. Zinc and iron deficiency were reported to be strongly correlated. The level of serum zinc was found to be significantly lower in the population with iron deficiency anaemia than in a healthy population.	[9]

**Table 5:** Chemical composition of chickpeas and lentils: raw and freeze-dried cooked (g/100 g) [80].

Legumes	Protein	Carbohydrate	Lipids	Ash	Crude Fibre	Insoluble Dietary Fibre	Soluble Dietary Fibre
Raw chickpeas	18.50 ± 1.74	54.00 ± 3.30	6.69 ± 0.56	3.15 ± 0.20	9.88 ± 2.11	13.90 ± 0.09	0.00 ± 0.00
Cooked chickpeas	21.30 ± 0.73	57.80 ± 2.11	6.73 ± 0.63	3.48 ± 0.03	8.50 ± 0.55	15.40 ± 0.18	0.00 ± 0.00
Raw lentils	20.60 ± 0.37	56.40 ± 4.08	2.15 ± 0.14	2.80 ± 0.15	6.83 ± 2.42	19.00 ± 1.27	1.44 ± 0.11
Cooked lentils	23.44 ± 0.64	61.80 ± 1.24	2.36 ± 0.13	3.12 ± 0.37	5.69 ± 0.33	21.40 ± 2.10	1.37 ± 0.52

**Table 6:** Mineral and vitamin composition of chickpea and lentil (mg/100 g) [81].

	Mineral Component				Vitamin					
Pulses	Ca	Mg	Fe	Zn	FA	Vit C	Vit B12	Vit D	Vit A	TCP
Chickpea	49	48	2.89	1.53	299.0	1.34	NA	NA	NA	12.9
Lentil	19	36	3.33	1.27	138.1	0.71	NA	NA	NA	5.64

Vit—Vitamin; FA = Folic acid; TCP—Tocopherol; NA—Not Available. The values are mean values for mature seeds of chickpeas and lentils, cooked and boiled without salt.

**Table 7:** Incorporation of probiotic in dry food matrices.

No	Food Matrix	LAB Species	Viability Cell Count (Initial to Final)	Storage Time and Condition	Notes	References
1	Savoury cereal bar	*Lactobacillus acidophilus*	10.3 log CFU/g–5.2 log CFU/g	120 d, 4 °C	-	[101]
		*Bifidobacterium animalis* subsp. *Lactis*	10.5 log CFU/g–3.1 log CFU/g	121 d, 4 °C	-	
2	Peanut butter	*Lactobacillus rhamnosus* GG.	10^7^ CFU/g to 10^6^ log CFU/g	48 w, 4 °C	-	[117]
3	Chocolate soufflé	*Lactobacillus reuteri* DSM 17938	7.4 log CFU/g	-	Probiotic coated in alginate beads with chitosan by spray drying	[118]
4	Pan bread	*Lactobacillus rhamnosus* GG.	from 7.57–8.98 log CFU/g to 6.55–6.91 log CFU/g	-	Viability cells in invitro digestion. Probiotic coated in sodium alginate and whey protein concentrate by air drying	[119]
5	Soy bar	*Lactobacillus acidophilus* LA-2	2.5 × 10^8^ CFU/g	8 w, 4 °C	Probiotics are microencapsulated with K-carrageenan and inulin. No significant reduction in viability reported	[120]
6	Milk and dark chocolate	*Lactobacillus acidophilus* NCFM^®^	8.7 log CFU/g–8.5 log CFU/g	180 d, 4 °C	-	[121]
		*Bifidobacterium lactis* HN019	8.13 log CFU/g–6.8 log CFU/g	120 d, 4 °C		
7	Dried apple	*Bifidobacterium bifidum*	9.5 log CFU/g to 5.28 log CFU/g	25 d, 4 °C	Probiotic is encapsulated with sodium alginate and carrageenan	[122]
8	Dried apple	*Lactobacillus paracasei*	from 7.42–7.99 log CFU/g to 6–7 log CFU/g	28 d, 4 °C	Apple slices impregnated with probiotic and dried with convectional of vacuum drying	[123]
9	Hamburger bun	*Lactobacillus casei* 431	7.4 × 10^8^ CFU/g–7.1 × 10^8^ CFU/g	4 d, 25 °C	Probiotic coated in alginate and starch beads	[124]
10	Fruit powder (apple, banana, and strawberry)	*Lactobacillus plantarum* 299v	from 8.2–9.3 log CFU/g to 7.3–8.6 log CFU/g	30 d, 4 °C	30% addition of probiotic to the fruit powder	[125]

## Data Availability

Not applicable.
